# What Shall We Do with Our Vice-Presidents?

**Published:** 1900-09

**Authors:** 


### WHAT SHALL WE DO WITH OUR VICE-PRESIDENTS?

Ten years ago our National Association, with its limited membership and circumscribed territory, found one Vice-President rather an ornamental official. To-day, in its broadened geographical lines, its widened sphere of usefulness, it has decided that it needs five. While there may be room for a difference of opinion as to whether we shall regard them from a purely geographical position, or the personal popularity in numerical order as to the number of votes received by each, or a stepping-stone to the Presidency of our national organization, surely with so many burning problems for us to solve, these positions should no longer be regarded as purely honorary ones, but they should be five great centres of influence and power for the better completion of our work. Our growth being so fast, our membership multiplying with such dangerous rapidity, our great country's geographical character, all point to the planning of certain aspects of our work that might well be done by those upon whom the Association's choice falls for these places.

What will our Association do to garner the harvest that awaits our gathering from such worthy laborers in the field of veterinary medicine?

